# Live-imaging of Bioengineered Cartilage Tissue using Multimodal Non-linear Molecular Imaging

**DOI:** 10.1038/s41598-019-41466-w

**Published:** 2019-04-03

**Authors:** Catarina Costa Moura, Konstantinos N. Bourdakos, Rahul S. Tare, Richard O. C. Oreffo, Sumeet Mahajan

**Affiliations:** 1 Institute for Life Sciences and Department of Chemistry, Highfield Campus, University of Southampton, SO17 1BJ Southampton, UK; 2 Bone and Joint Research Group, Centre for Human Development, Stem Cells and Regeneration, Institute of Developmental Sciences, Faculty of Medicine, University of Southampton, SO16 6YD Southampton, UK; 3 Mechanical Engineering Department, Faculty of Engineering and the Environment, Highfield Campus, University of Southampton, SO17 1BJ Southampton, UK

## Abstract

Coherent anti-Stokes Raman scattering (CARS) and second harmonic generation (SHG) are non-linear techniques that allow label-free, non-destructive and non-invasive imaging for cellular and tissue analysis. Although live-imaging studies have been performed previously, concerns that they do not cause any changes at the molecular level in sensitive biological samples have not been addressed. This is important especially for stem cell differentiation and tissue engineering, if CARS/SHG microscopy is to be used as a non-invasive, label-free tool for assessment of the developing neo-tissue. In this work, we monitored the differentiation of human fetal-femur derived skeletal cells into cartilage in three-dimensional cultures using CARS and SHG microscopy and demonstrate the live-imaging of the same developing neo-tissue over time. Our work conclusively establishes that non-linear label-free imaging does not alter the phenotype or the gene expression at the different stages of differentiation and has no adverse effect on human skeletal cell growth and behaviour. Additionally, we show that CARS microscopy allows imaging of different molecules of interest, including lipids, proteins and glycosaminoglycans, in the bioengineered neo-cartilage. These studies demonstrate the label-free and truly non-invasive nature of live CARS and SHG imaging and their value and translation potential in skeletal research, regeneration medicine and tissue engineering.

## Introduction

Tissue engineering has been described as the application of scientific methods to produce ‘spare parts’ of the body for replacement of damaged or lost organs^1–3^. Skeletal tissue engineering seeks to address the growing need for skeletal tissue augmentation or repair through the generation of functional skeletal tissue by the recapitulation of stem cell developmental processes. A major challenge in Orthopaedics is the regeneration of articular cartilage and the application of cell-based restorative and reparative surgical techniques for articular cartilage repair^4,5^. Human skeletal cell populations offer significant potential as a cell source for tissue engineering applications, and in particular for skeletal tissue regeneration strategies^6,7^. The development of appropriate tools to non-invasively follow the development of skeletal cells and the formation of engineered neo-cartilage in real-time and non-destructively is crucial and remains, to date, an unmet goal.

Coherent anti-Stokes Raman scattering (CARS) combined with microscopy is a powerful chemical imaging technique that maps the distribution of molecules in biological systems in their native state, without the need for an external label (such as stains or fluorophores)^8^. The label-free nature of CARS microscopy, together with its inherent three-dimensional imaging capability^9^, presents an exciting imaging tool for biomedical and clinical applications. Given sample preparation and processing are not required, live-imaging using CARS microscopes has become a reality^10–12^. As with all optical techniques, power and exposure to light need to be within a threshold to prevent any cell damage and phototoxic effects. However, with CARS microscopy, a number of questions remain as to whether: (i) live-imaging using CARS microscopy is fully non-invasive; (ii) cell development remains unaltered; and (iii) the cells remain viable and robust for further use in clinical applications following live-imaging with CARS microscopy. Studies have reported on thresholds of photo-induced cell damage by CARS microscopy, commonly by visualising direct cell morphological changes^13^, and detecting formation of apoptotic membrane protrusions^14^, or by analysing and comparing nuclear staining between damaged and non-damaged cells after laser exposure^15^. The induced damage and changes are obvious at the levels of damage thresholds. Furthermore, simultaneously with CARS, second harmonic generation (SHG), a well-established technique that allows imaging of collagen fibres in tissues, can be carried out with appropriate laser sources^16^. For both non-linear techniques, CARS and SHG, given that relatively high peak powers are used, it is therefore necessary to establish that no subtle changes are induced that are detrimental to the biological system under study (even if the laser powers are within damage thresholds).

Previous studies have shown that Raman spectroscopy^17,18^ and CARS/SHG^19–22^ could be used for two- and three-dimensional cell cultures, but currently there are no known studies detailing live cell state or cell development over time using non-linear imaging. Critically, there has been no investigation, to date, detailing the potential biological effects on using non-linear imaging techniques such as CARS and SHG on live tissue when the excitation powers are within damage thresholds. This is essential to establish CARS and related non-linear imaging techniques as mainstream analytical or assessment tools in biomedicine and, more specifically, in skeletal repair and regeneration strategies. The application of robust, real-time, temporal, non-invasive imaging is relevant for tissue engineering, in particular, to ensure the absence of tissue and cell deterioration over time and to investigate appropriate tissue development at the molecular-level. The current study examines these issues with analysis of the development of human fetal femur-derived skeletal cells into cartilage, and sets out to conclusively establish, through gene expression analysis and concomitant imaging, that the non-linear imaging process itself does not have any observed effect during cell differentiation (carried out over 21 days). Furthermore, critical in longitudinal cell and tissue differentiation studies is the judicious selection of appropriate targets/markers. Lipids remain the molecule of choice in most studies for imaging using CARS, given the role of lipids in metabolism and their strong Raman signal due to CH-stretching vibrations^23^. More recently, the potential of CARS microscopy for imaging other relevant biological molecules such as phosphate in hydroxyapatite^24,25^, or nucleic acids and proteins^25,26^, has attracted significant interest. Here we demonstrate the ability of CARS microscopy to image relevant molecules, namely proteins and glycosaminoglycans, in the bioengineered cartilage tissue. Our work thus establishes the translation potential of label-free multimodal non-linear imaging approaches for biomedicine and paves the way for their application to cell-based therapies, human skeletal regeneration research and tissue engineering.

## Results and Discussion

**Figure 1:**
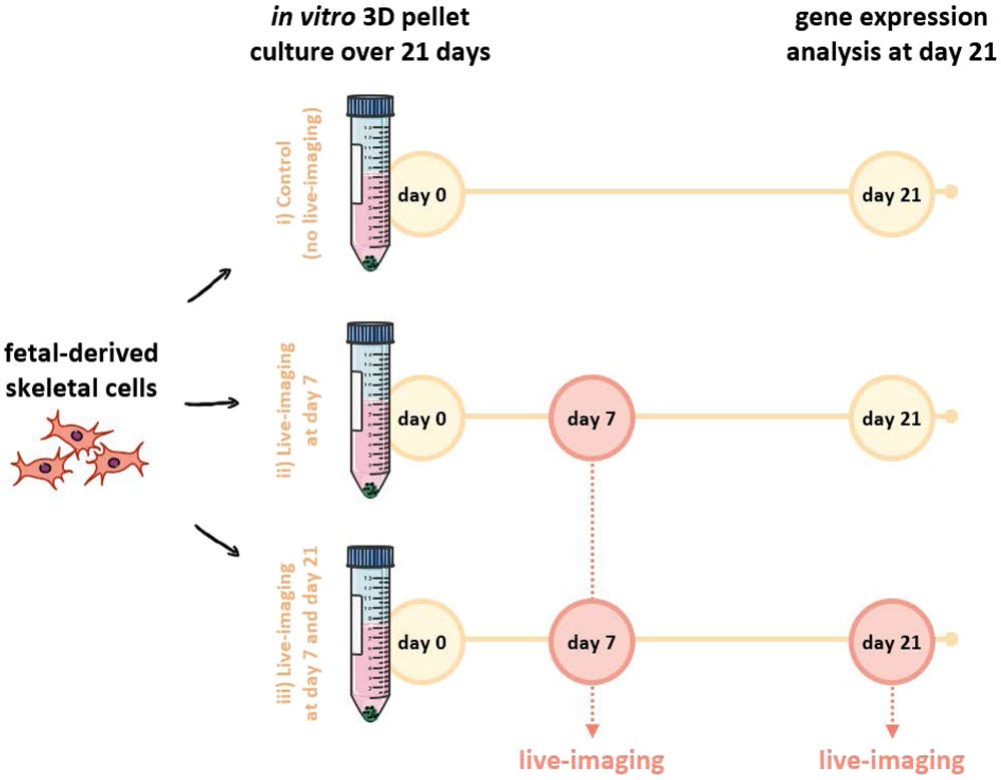
Schematic diagram of the experimental design. Human fetal femur-derived skeletal cells were cultured in chondrogenic medium in an *in vitro* three-dimensional pellet culture system for 21 days. From the same fetal sample: (i) cells were cultured over 21 days in the absence of live-imaging (control); (ii) cells were cultured over 21 days and live-imaging performed at day 7; and (iii) cells were cultured over 21 days and live-imaging performed at day 7 and day 21. The expression of skeletal lineage-specific genes after 21 days in chondrogenic culture was analysed for all three conditions.

Human fetal femur-derived skeletal cells were differentiated into cartilage and analysed using live-CARS and SHG microscopy. The culture of human fetal skeletal cells was carried out in an *in vitro* three-dimensional pellet culture system for 21 days in chondrogenic medium to differentiate the skeletal cells into chondrocytes and to generate neo-cartilage (Fig. 1). Skeletal cell aggregation was carried out in chondrogenic media to form cartilaginous pellets. The initiation and formation of the extracellular matrix imbues the bioengineered construct with structural integrity. In order to establish that live-imaging by CARS and SHG does not affect the development of the bioengineered cartilage the following experiment was designed. Three different conditions using human skeletal cells derived from the same fetal sample were examined: (i) cells cultured over 21 days in absence of live-imaging (control); (ii) cells cultured over 21 days and live-imaging performed at day 7; and, (iii) cells cultured over 21 days and live-imaging performed at day 7 and day 21 (Fig. 1). Although no differences are expected between the two former conditions, fetal skeletal cells were imaged at day 21 under condition (iii) to confirm cell integrity. Fetal skeletal cells were imaged in real-time under identical culture and chondrogenic environment as the control. Skeletal lineage-specific gene expression was analysed in all three conditions.

**Figure 2:**
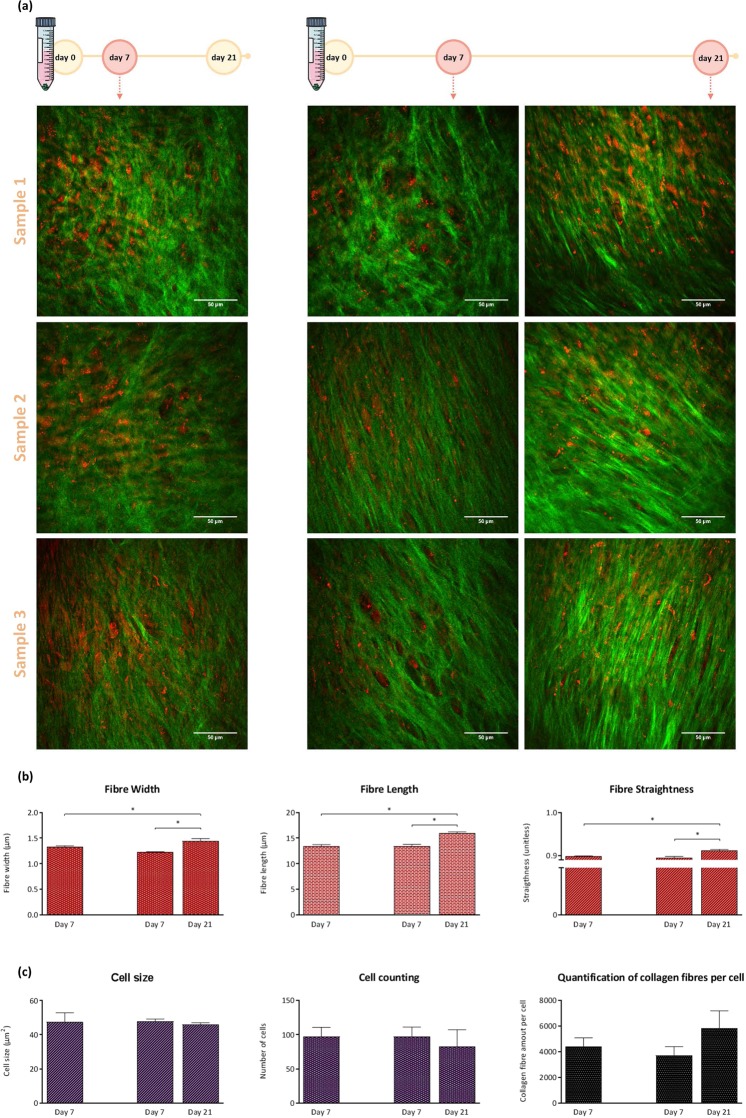
(**a**) Label-free live-imaging at day 7 and day 21 of fetal (human) femur-derived skeletal cell pellets cultured in chondrogenic media for 21 days. SHG imaged fibres of collagen (green) and lipid droplets (red) within the pellet of the neo-cartilage were imaged by CARS. Scale bars correspond to 50 µm. (**b**) Quantification of the width, length and straightness of collagen fibres. CT-FIRE was used to extract these details from the SHG images of collagen fibres. (**c**) Fiji was used to analyse CARS images to quantity the number of cells and their sizes for each cartilage pellet. Quantification was carried out to evaluate the amount of collagen fibres found per number of cells in the neo-cartilage tissue using both CARS and SHG data. Average data from 3 independent samples is shown where error bars indicate the observed standard deviation. The Mann-Whitney test was used to calculate the level of significance at *p-value < 0.05.

Live-imaging of chondrogenic differentiation of skeletal cell populations was performed using a label-free, multimodal, non-linear imaging platform, combining CARS and SHG microscopy (Fig. 2a). A 2 picosecond pulsed laser system was used for simultaneous CARS and SHG imaging to get the benefit of high spectral resolution (~10 cm^−1^) and efficiency in CARS without compromising much on signal generation with SHG. The laser power required for the live-imaging procedure to be used in fetal femur-derived skeletal cells without damaging the pellet structure was found to be approximately 120 mW, after a thorough protocol optimisation. SHG enabled imaging of collagen fibres within the 3D cartilage pellet, providing a comprehensive structural information on the collagen fibre network without using labels. CARS microscopy allowed the visualisation of the lipid distribution during chondrogenesis. At different time-points, all samples were equivalent and presented comparable collagen and lipid patterns. Quantification of the image analysis data sets (Fig. 2b) demonstrated that collagen fibres increase significantly in width, length and straightness, from day 7 to day 21 of chondrogenic culture. No statistically significant differences in cell size and cell number between the different days of culture, at the same field of view, were observed (Fig. 2c). Image analysis enabled measurement of the amount of collagen fibres present in the same region of interest, to quantify collagen and the number of cells during development of the neo-cartilage. An increase from day 7 to day 21 was observed signifying the net balance between the proliferation of fetal skeletal cells and the deposition of collagen (Fig. 2c).

**Figure 3:**
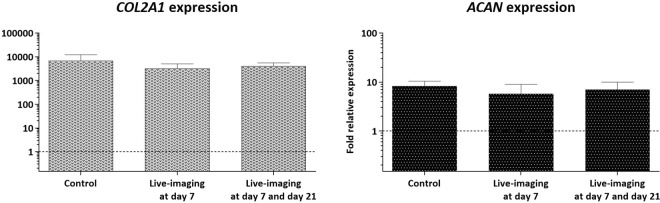
Expression of *COL2A1* and *ACAN* in skeletal cells derived from human fetal femurs chondrogenic culture at day 21, including control (cells cultured with no live-imaging), cells cultured over 21 days and live-imaging performed at day 7, and cells cultured over 21 days and live-imaging performed at both days 7 and 21. Normalisation was carried out with respect to *ACTB* to obtain relative gene expression and the gene expression on day 0 was assigned a value of 1 (as indicated by the dotted line). Average data from 3 independent samples is shown where error bars indicate the observed standard deviation. The Mann-Whitney test was used to calculate the level of significance at *p-value < 0.05.

Gene expression analysis using reverse transcription quantitative polymerase chain reaction (RT-qPCR) to compare the three different conditions is detailed in Fig. 3. The characteristic chondrogenic genes *COL2A1* and *ACAN* were up-regulated. These encode the α-chain of hyaline cartilage-specific Type II collagen and the main proteoglycan in cartilage, respectively, in fetal skeletal cells following 21 days of chondrogenic culture (Fig. 3), as previously demonstrated^27,28^. *COL10A1*, which encodes the α-chain of Type X collagen expressed by hypertrophic chondrocytes, and *SOX9*, the chondrogenic transcription factor, were negligibly expressed after 21 days of culture (gene amplification only detected beyond 30 cycles). Furthermore, a down-regulation of characteristic osteogenic (*ALPL*) and adipogenic (*PPARG* and *FABP4*) genes was observed after 21 days of differentiation in chondrogenic culture (Supplementary Fig. 1).

Crucially, analysis across the three human fetal skeletal cell culture conditions (control, live-imaging at day 7, and live-imaging at day 7 and 21) indicated no significant difference in the expression levels for chondrogenic genes. Comparison against the control demonstrated that the live-imaging procedure with CARS and SHG microscopy, either imaged at one single early time-point (day 7) or two distinct time-points (day 7 and day 21), did not restrict or affect chondrogenic differentiation and development of the cartilage tissue at the molecular level.

The above results on molecular expression allow us to conclude that for the non-linear imaging techniques used here the lack of ‘morphological damage’ observed in the developing neo-cartilage tissue is correlated to the lack of ‘molecular damage’ and ‘growth impairment’ observed. We note that this may be due to the specific methodology and procedures we used: 120 mW of a single scan (pixel dwell time of ~14.3 µs and power density of <6 MW/cm^2^) as well as the fact that the tissue was always in sterile conditions and the culture media was immediately replenished after each scan. Our observation regarding the lack of morphological damage observed is consistent with findings of other groups, who observed photodamage after several tens of scans at such powers^14,29^. Thus, label-free multimodal imaging offers a singular non-invasive tool for stem cell biologists to study tissue repair and regeneration in a live and a non-destructive manner.

**Figure 4:**
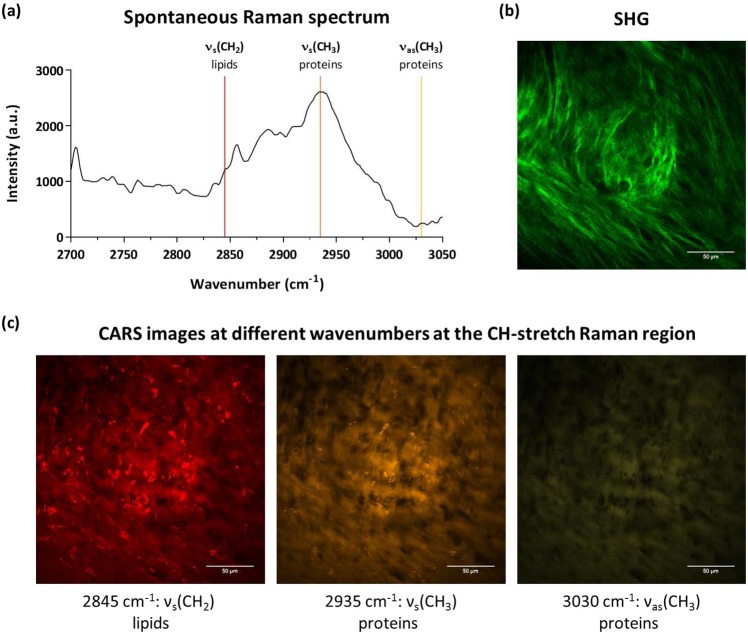
Human fetal femur-derived skeletal cells were cultured as a three-dimensional pellet over 21 days in chondrogenic media to generate cartilage tissue. (**a**) Raman spectrum in the CH-stretch region. The three marked bands were targeted for CARS imaging. (**b**) SHG images fibres of collagen (green) in the bioengineered cartilage tissue. (**c**) CARS images of the bioengineered cartilage tissue at the CH-stretch region: 2845 cm^−1^ (ν_s_(CH_2_)), 2935 cm^−1^ (ν_s_(CH_3_)), and 3030 cm^−1^ (ν_as_(CH_3_)). Scale bars correspond to 50 µm.

After monitoring the differentiation of skeletal cells into cartilage in three-dimensional cultures for 21 days, the potential of the multimodal label-free system to image other relevant molecules in the cartilage tissue was investigated. CARS microscopy is commonly applied to image molecules in the CH-stretch region of the Raman spectrum (2840–3000 cm^−1^), such as lipids and cell membranes, important in tissue and cellular analysis^30^. The Raman spectrum of the cartilage tissue in the CH-stretch region was acquired (Fig. 4a). Images from lipids in the cell pellets were captured by targeting the Raman CH stretching mode at 2845 cm^1^ (CH_2_ symmetric stretch)^23,31^, and the SHG signal was simultaneously acquired (Fig. 4b and c). The pump beam used for CARS imaging also served as the SHG excitation source (with a separate detection channel). Furthermore, label-free images of proteins within the cartilage construct (Fig. 4c) were collected by targeting the vibrational modes at 2935 cm^−1^ (CH_3_ symmetric stretch) and 3030 cm^−1^ (CH_3_ asymmetric stretch)^31^. This was performed sequentially by tuning the pump beam to target the corresponding vibrational frequencies. Similar structures can be observed by targeting different vibrational modes (Fig. 4). Although the 2935 cm^−1^ vibration mode is commonly assigned to proteins, lipids also have CH_3_ moieties that will be detected using CARS microscopy. As CARS provides the chemical distribution of a particular vibrational mode, different molecules at a particular frequency can be observed. Specifically, the multimodal imaging system used in this work has a spectral resolution of ~10 cm^−1^ and there is no ‘bleed-through’ in the different channels.

**Figure 5:**
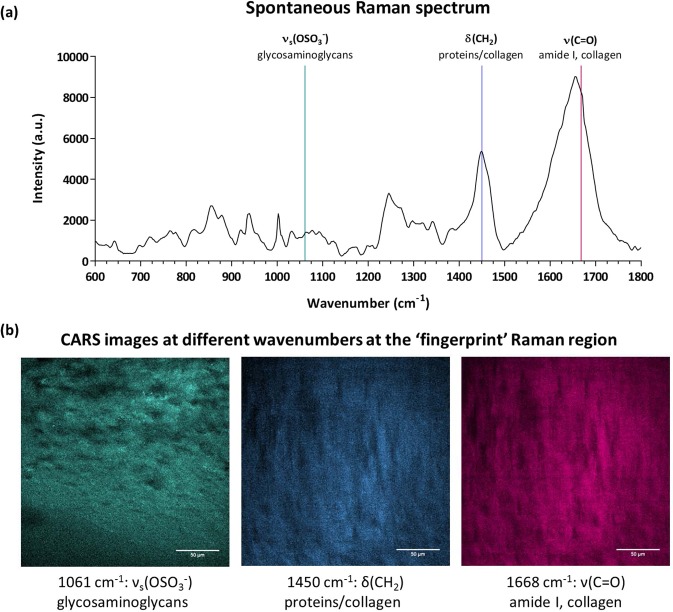
Human fetal femur-derived skeletal cells were cultured as a three-dimensional pellet over 21 days in chondrogenic media to generate cartilage tissue. (**a**) Raman spectrum in the ‘fingerprint’ region. The three marked bands were targeted for CARS imaging. (**b**) CARS images acquired at the ‘fingerprint’ vibrations of: 1061 cm^−1^ (ν_s_(OSO_3_^−^)), 1450 cm^−1^ (δ(CH_2_)), and 1668 cm^−1^ (ν(C=O)). Scale bars correspond to 50 µm.

Although extensive work has been undertaken employing CARS imaging in the CH stretch region of the Raman spectrum, imaging on the spectral region between 800 cm^−1^ and 1800 cm^−1^ using CARS microscopes has received less attention. This spectral Raman region, the so-called ‘fingerprint’ region, is rich in biochemical information including chemical functional groups related to tissue proteins, lipids, glycogen and nucleic acids (Fig. 5a). Articular cartilage consists primarily of extracellular matrix, such as collagens, proteoglycans and non-collagenous proteins, and a sparse population of chondrocytes. Proteoglycans have an important role in the cartilage matrix and are composed of a protein core and one or more glycosaminoglycan chains, which hold negatively charged carboxylate or sulphate groups^32^. The Raman band at 1061 cm^−1^ is typical from glycosaminoglycans with sulphate groups (OSO^3−^ symmetric stretch)^33^, and by targeting this vibrational mode glycosaminoglycans can be imaged using CARS microscopy (Fig. 5b). Additionally, the amide I band at 1668 cm^−1^ is mainly assigned to collagen^33–35^, and CARS signal from both fibrillar and non-fibrillar collagen can be observed in Fig. 5b. The Raman CH vibrational mode at 1450 cm^1^ (CH_2_/CH_3_) is related to collagen/proteins^33–35^, and although slightly different, one can observe the similarities between the 1450 cm^1^ and 1668 cm^−1^ signals. CARS images at different vibrational frequencies acquired from replicate samples are shown in Supplementary Fig. 2.

**Figure 6:**
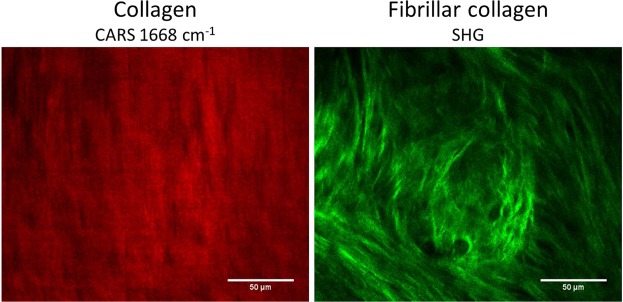
Coherent anti-Stokes Raman scattering (CARS) signal at 1668 cm^−1^, mainly assigned to collagen, and SHG signal, revealing fibrillar collagen on the bioengineered cartilage tissue.

Interestingly, CARS imaging at the 1668 cm^−1^ Raman band displayed a distinct and different signal of collagen compared to SHG. In fact, while CARS is responsive to the molecular structure and chemical composition, in this case proteins that are predominantly collagens, SHG is sensitive to the supermolecular crystalline structure of collagen^36,37^. Cartilage predominantly is composed of collagen Type II, which is fibrillar and hence is imaged by SHG in this case. However, not all collagen types are fibrillar. Therefore, combined multimodal imaging could offer insight with regards to the distribution of fibrillar and non-fibrillar collagen and their respective types^37–39^. In this case, the home-built label-free multimodal system allowed acquisition of information on collagen by mapping the CARS signal at 1668 cm^−1^ band and on fibrillar collagen by analysing the SHG signal, highlighting the potential of a multimodal label-free imaging system equipped with non-linear techniques such as CARS and SHG as a powerful monitoring tool in tissue engineering (Fig. 6).

## Conclusions

In summary, the current studies have investigated the use of label-free non-linear imaging techniques, namely CARS and SHG microscopy, in live human fetal femur-derived skeletal cells differentiated into chondrogenic cultures. 2 picosecond pulsed lasers were used to acquire CARS and SHG images. The results demonstrate that under damage thresholds, label-free live-imaging had a negligible effect on skeletal cell (human fetal) differentiation and development into cartilage-tissue at the level of molecules, as confirmed by gene expression analysis. The live-imaging allowed monitoring of fetal femur-derived skeletal cells without compromising cell differentiation and cartilage development. Furthermore, these studies demonstrate that CARS microscopy allows imaging of additional key molecules of interest such as lipids, proteins and glycosaminoglycans in bioengineered cartilage tissue. This study indicates that multimodal imaging with non-linear techniques such as CARS and SHG offer new approaches for clinical translation in assessment of regenerated skeletal tissues, and are ready for widespread implementation by biomedical scientists. Overall, our demonstration of live-imaging ability to dynamically follow the formation of neo-(regenerated)-tissues in real-time using non-invasive techniques paves the way for exciting opportunities in the design and development of innovative tissue engineering solutions for hard and soft tissues.

## Methods

### Fetal skeletal cell isolation and culture

Human fetal tissue were obtained after termination of pregnancy procedure and with informed consent of patients following the guidelines issued by the Polkinghome Report as per the approval (LREC 296100) from the Southampton and South West Hampshire Local Research Ethics Committee. The fetal samples were at the end of Carnegie stage 23 (approximately 60 days post conception) and were isolated from the lower limbs. Surrounding skeletal muscle and connective tissue were removed. Segments were cut from each sample, treated and cultured as in our previous publications^27^. Briefly, cells were harvested at 85% confluence using 0.025% (w/v) trypsin/EDTA with 0.05% glucose for 5 minutes at 37 °C and cryopreserved in 10% (v/v) dimethyl sulfoxide (DMSO) in fetal calf serum (FCS). Fetal skeletal cells were thawed and cultured in α-MEM supplemented with 10% FCS, 100 U/mL penicillin and 100 mg/mL streptomycin before each experiment, as previously described^27,40^.

### Chondrogenic differentiation

Human fetal-femur derived skeletal cells were re-suspended in chondrogenic media with no serum (α-MEM supplemented with 100 U/mL penicillin and 100 mg/mL streptomycin, 100 μM ascorbate-2-phosphate, 10 ng/mL TGFβ3, 10 nM dexamethasone and 1x ITS liquid media supplement [10 µg/mL recombinant human insulin, 5.5 µg/mL human transferrin and 5 ng/mL sodium selenite]) at a cell density of 3 × 10^5^ cells/mL, as previously described^27^. After a second centrifugation, the resulting cell pellet was maintained in a humidified chamber at 37 °C and 5% CO_2_ for 21 days. Chondrogenic media was restocked every 2–3 days^28^.

### Live imaging

Human fetal-femur derived skeletal cell pellets cultured for 7 and 21 days were washed with phosphate buffered saline (PBS) and placed in a sterile coverslip cell chamber for live-imaging (Supplementary Fig. 3). Label-free multimodal imaging was performed using a home-built laser scanning system (Supplementary Fig. 4), which allows simultaneous image acquisition of CARS and SHG, using ScanImage 5.1 (Vidrio Technologies)^41^. For CARS imaging, a 2 picosecond APE laser system was used, as previously described^27^. The CH-stretch mode at 2845 cm^−1^ was targeted by tuning the OPO to 797.8 nm. The SHG signal was acquired using the same laser source at 797.8 nm. Each sample was imaged using a 20x/0.75 NA water immersion objective, with 14.5 ms per line period for a 1024 × 1024-pixel image. The total incident power on the sample was approximately 120 mW (80 mW from pump and 40 mW from Stokes). A single scan was taken at one area of the sample. The power density calculated is <6 MW/cm^2^ at the sample. After live-imaging, serum-free chondrogenic media was replenished, and human fetal-femur derived skeletal cell pellets were maintained in an incubator at 37 °C and 5% CO_2_ until ready for gene expression analysis.

### Multispectral imaging with CARS

For CARS imaging at different wavenumbers fixed human fetal-femur derived skeletal cell pellets were placed in a sterile coverslip cell chamber on the same setup as above. The different vibrational modes, 1061 cm^−1^, 1450 cm^−1^, 1668 cm^−1^, 2845 cm^−1^, 2935 cm^−1^ and 3030 cm^−1^, were targeted by tuning the OPO to 930.2 nm, 897.7 nm, 880.4 nm, 797.8 nm, 792.1 nm, and 786.2 nm, respectively. Supplementary Table 1 shows the required modifications in the multimodal label-free imaging set up to acquire CARS images at different wavenumbers.

### Gene expression analysis

#### RNA extraction and cDNA synthesis

Bioengineered cartilage tissue samples were disrupted and homogenised in a lysis buffer (lysis buffer TX from Bioline, England with 1% (v/v) β-mercaptoethanol) after 21 days of chondrogenic culture. The Bioline Isolate II RNA/DNA/Protein kit was used to isolate total RNA, following the manufacturer’s instructions. Dilutions were prepared to normalise the amount of RNA for each sample in the experiment. cDNA was synthesised using TaqMan™ Reverse Transcription Reagents from Applied Biosystems™, according to the manufacturer’s instructions.

#### Reverse transcription quantitative polymerase chain reaction (qPCR)

An ABI Prism 7500 detection system (Applied Biosystems) was used to quantify relative quantification of gene expression, as previously described^27^. Supplementary Table 2 shows the primers used for qPCR. The experiment was performed using 2 µL of cDNA, 17.5 µL of GoTaq qPCR Master Mix (Promega, Madison, WI, USA) and 1.5 µL of each primer (5 µM). All reactions were performed in triplicate and included a negative control (water in place of cDNA sample). All data were normalised to β-actin expression (*ACTB*) and compared to the expression values of each gene at day 0.

### Raman spectroscopy

Human fetal-femur derived skeletal cell pellets cultured for 21 days were fixed in 4% (v/v) formaldehyde solution^27^. Raman spectra were obtained using a Renishaw® inVia Raman microscope in combination with WiRE 3.4 software, with a 633 nm laser and a 20x/0.5 NA water immersion objective. For each spectrum, 3 accumulations were collected using 6 mW laser power at the sample, an exposure time of 60 seconds and a 1200 lines per mm grating. Final spectra were pre-processed using IRootLab^27,42^.

### Image processing and analysis

Collagen fibres in SHG images were extracted and quantified using CT-FIRE^27,43^. All images were processed using Fiji. The size and number of cells, as well as the ratio between the amount of collagen fibres to number of cells per area of interest were quantified. CARS images were processed and analysed as previously described^27^.

### Statistical analysis

All experiments were performed using three different human fetal samples. GraphPad Prism 7 (San Diego, CA, USA) was used to prepare all graphs., and IBM^®^ SPSS® Statistics version 21.0 (IBM Corporation, Armonk, NY, USA) to perform statistical analysis. The Mann-Whitney U-test was performed to compare two independent groups. Differences were considered to be statistically significant at  *p*-value ≤ 0.05.

## Supplementary information

Supplementary information

## References

[1] Rose FR, Oreffo RO (2002). Bone tissue engineering: hope vs hype. Biochemical and biophysical research communications.

[2] Bianco P, Robey PG (2001). Stem cells in tissue engineering. Nature.

[3] Vacanti, J. P. & Langer, R. Tissue engineering: the design and fabrication of living replacement devices for surgical reconstruction and transplantation. *The Lancet* **354**, S32–S34, 10.1016/S0140-6736(99)90247-7. 10.1016/s0140-6736(99)90247-7 10437854

[4] Shimomura K (2018). Scaffold-free tissue engineering for injured joint surface restoration. Journal of experimental orthopaedics.

[5] Yang Y (2018). Mesenchymal Stem Cell-Derived Extracellular Matrix Enhances Chondrogenic Phenotype of and Cartilage Formation by Encapsulated Chondrocytes *in vitro* and *in vivo*. Acta biomaterialia.

[6] Cheung KS (2014). MicroRNA-146a regulates human foetal femur derived skeletal stem cell differentiation by down-regulating SMAD2 and SMAD3. PloS one.

[7] Dawson JI, Kanczler J, Tare R, Kassem M, Oreffo ROC (2014). Concise Review: Bridging the Gap: Bone Regeneration Using Skeletal Stem Cell-Based Strategies—Where Are We Now. Stem Cells.

[8] Moura, C. C., Tare, R. S., Oreffo, R. O. C. & Mahajan, S. Raman spectroscopy and coherent anti-Stokes Raman scattering imaging: prospective tools for monitoring skeletal cells and skeletal regeneration. *Journal of The Royal Society Interface* **13**, 10.1098/rsif.2016.0182 (2016). 10.1098/rsif.2016.0182 PMC4892269 27170652

[9] Zumbusch A, Holtom GR, Xie XS (1999). Three-Dimensional Vibrational Imaging by Coherent Anti-Stokes Raman Scattering. Physical Review Letters.

[10] Khmaladze A (2014). Hyperspectral imaging and characterization of live cells by broadband coherent anti-Stokes Raman scattering (CARS) microscopy with singular value decomposition (SVD) analysis. Applied spectroscopy.

[11] Bradley J (2016). Quantitative imaging of lipids in live mouse oocytes and early embryos using CARS microscopy. Development (Cambridge, England).

[12] Nan X, Cheng JX, Xie XS (2003). Vibrational imaging of lipid droplets in live fibroblast cells with coherent anti-Stokes Raman scattering microscopy. Journal of lipid research.

[13] Nan X, Potma EO, Xie XS (2006). Nonperturbative chemical imaging of organelle transport in living cells with coherent anti-stokes Raman scattering microscopy. Biophysical journal.

[14] Fu Y, Wang H, Shi R, Cheng JX (2006). Characterization of photodamage in coherent anti-Stokes Raman scattering microscopy. Optics express.

[15] Minamikawa T (2017). Photo-Induced Cell Damage Analysis for Single- and Multifocus Coherent Anti-Stokes Raman Scattering. Microscopy. Journal of Spectroscopy.

[16] Campagnola PJ, Loew LM (2003). Second-harmonic imaging microscopy for visualizing biomolecular arrays in cells, tissues and organisms. Nature biotechnology.

[17] Kallepitis C (2017). Quantitative volumetric Raman imaging of three dimensional cell cultures. Nature Communications.

[18] Smith SJ, Emery R, Pitsillides A, Clarkin CE, Mahajan S (2017). Detection of early osteogenic commitment in primary cells using Raman spectroscopy. Analyst.

[19] Mortati L, Divieto C, Sassi MP (2012). CARS and SHG microscopy to follow collagen production in living human corneal fibroblasts and mesenchymal stem cells in fibrin hydrogel 3D cultures. Journal of Raman Spectroscopy.

[20] Hofemeier AD (2016). Label-free nonlinear optical microscopy detects early markers for osteogenic differentiation of human stem cells. Scientific reports.

[21] Xu X (2013). Multimodal non-linear optical imaging for label-free differentiation of lung cancerous lesions from normal and desmoplastic tissues. Biomedical Optics Express.

[22] Lee HS (2006). Imaging human bone marrow stem cell morphogenesis in polyglycolic acid scaffold by multiphoton microscopy. Tissue engineering.

[23] Smus JP (2015). Tracking adipogenic differentiation of skeletal stem cells by label-free chemically selective imaging. Chemical Science.

[24] Pegoraro AF, Slepkov AD, Ridsdale A, Moffatt DJ, Stolow A (2014). Hyperspectral multimodal CARS microscopy in the fingerprint region. Journal of biophotonics.

[25] Downes A, Mouras R, Bagnaninchi P, Elfick A (2011). Raman spectroscopy and CARS microscopy of stem cells and their derivatives(). Journal of Raman spectroscopy: JRS.

[26] Camp CH (2014). High-Speed Coherent Raman Fingerprint Imaging of Biological Tissues. Nature photonics.

[27] Costa Moura C (2018). Quantitative temporal interrogation in 3D of bioengineered human cartilage using multimodal label-free imaging. Integrative Biology.

[28] Tare RS, Howard D, Pound JC, Roach HI, Oreffo RO (2005). Tissue engineering strategies for cartilage generation–micromass and three dimensional cultures using human chondrocytes and a continuous cell line. Biochemical and biophysical research communications.

[29] Galli R (2014). Intrinsic indicator of photodamage during label-free multiphoton microscopy of cells and tissues. PloS one.

[30] Parekh SH, Lee YJ, Aamer KA, Cicerone MT (2010). Label-Free Cellular Imaging by Broadband Coherent Anti-Stokes Raman Scattering Microscopy. Biophysical journal.

[31] Long DA (2004). Infrared and Raman characteristic group frequencies. Tables and charts George Socrates John Wiley and Sons, Ltd, Chichester, Third Edition, 2001. Price £135. Journal of Raman Spectroscopy.

[32] Buckwalter JA, Mankin HJ, Grodzinsky AJ (2005). Articular cartilage and osteoarthritis. Instructional course lectures.

[33] Bonifacio A (2010). Chemical imaging of articular cartilage sections with Raman mapping, employing uni- and multi-variate methods for data analysis. Analyst.

[34] Gasior-Glogowska M, Komorowska M, Hanuza J, Ptak M, Kobielarz M (2010). Structural alteration of collagen fibres–spectroscopic and mechanical studies. Acta of bioengineering and biomechanics.

[35] Alebrahim MA, Krafft C, Popp J (2015). Raman imaging to study structural and chemical features of the dentin enamel junction. IOP Conference Series: Materials Science and Engineering.

[36] Camp CHJr, Cicerone MT (2015). Chemically sensitive bioimaging with coherent Raman scattering. Nature photonics.

[37] Chen X, Nadiarynkh O, Plotnikov S, Campagnola PJ (2012). Second harmonic generation microscopy for quantitative analysis of collagen fibrillar structure. Nature Protocols.

[38] Su P-J (2010). The discrimination of type I and type II collagen and the label-free imaging of engineered cartilage tissue. Biomaterials.

[39] Mouw JK, Ou G, Weaver VM (2014). Extracellular matrix assembly: a multiscale deconstruction. Nat Rev Mol Cell Biol.

[40] Mirmalek-Sani SH (2006). Characterization and multipotentiality of human fetal femur-derived cells: implications for skeletal tissue regeneration. Stem Cells.

[41] Pologruto TA, Sabatini BL, Svoboda K (2003). ScanImage: flexible software for operating laser scanning microscopes. Biomedical engineering online.

[42] Trevisan J, Angelov PP, Scott AD, Carmichael PL, Martin FL (2013). IRootLab: a free and open-source MATLAB toolbox for vibrational biospectroscopy data analysis. Bioinformatics.

[43] Bredfeldt JS (2014). Computational segmentation of collagen fibers from second-harmonic generation images of breast cancer. Journal of biomedical optics.

