# Supplementary material for: Live-imaging of Bioengineered Cartilage Tissue using Multimodal Non-linear Molecular Imaging
Source: Sci Rep. 2019 Apr 3;9:5561. doi: 10.1038/s41598-019-41466-w (PMC6447547; doi:10.1038/s41598-019-41466-w)
Supplement: Supplementary file 1 — Supplementary information [file 41598_2019_41466_MOESM1_ESM.pdf]

Supplementary Information

Live-imaging of Bioengineered Cartilage Tissue using Multimodal Non-linear Molecular Imaging

Catarina Costa Moura[a,b], Konstantinos N. Bourdakos[a], Rahul S. Tare[b,c], Richard O. C. Oreffo[b*], Sumeet Mahajan[a*]

[a]Department of Chemistry and Institute for Life Sciences, Highfield Campus, University of Southampton, Southampton SO17 1BJ, UK. *E-mail: s.mahajan@soton.ac.uk

[b]Bone and Joint Research Group, Centre for Human Development, Stem Cells and Regeneration, Institute of Developmental Sciences, Faculty of Medicine, University of Southampton, Southampton SO16 6YD, UK. *E-mail: richard.oreffo@soton.ac.uk

[c]Mechanical Engineering Department, Faculty of Engineering and the Environment, Highfield Campus, University of Southampton, Southampton SO17 1BJ, UK.

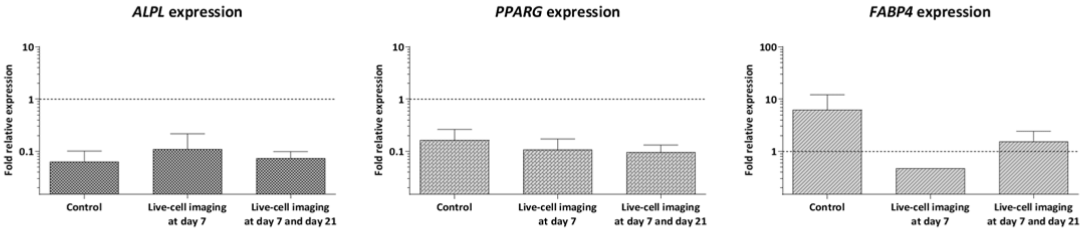

Supplementary Figure 1 – Expression of *ALPL*, *PPARg* and *FABP4* in human fetal femur-derived skeletal cells cultured in chondrogenic media at day 21, including control (cells cultured with no live-cell imaging), cells cultured over 21 days and live-cell imaging performed at day 7, and cells cultured over 21 days and live-cell imaging performed at both days 7 and 21. Relative gene expression was normalised to *ACTB*, and values for gene expression on day 0 were set to one (dotted line). For the three genes, gene amplification was not detected in some samples and the values were undetermined. Average of three independent fetal samples; error bars represent standard deviation. $P>0.05$ calculated using Mann-Whitney test.

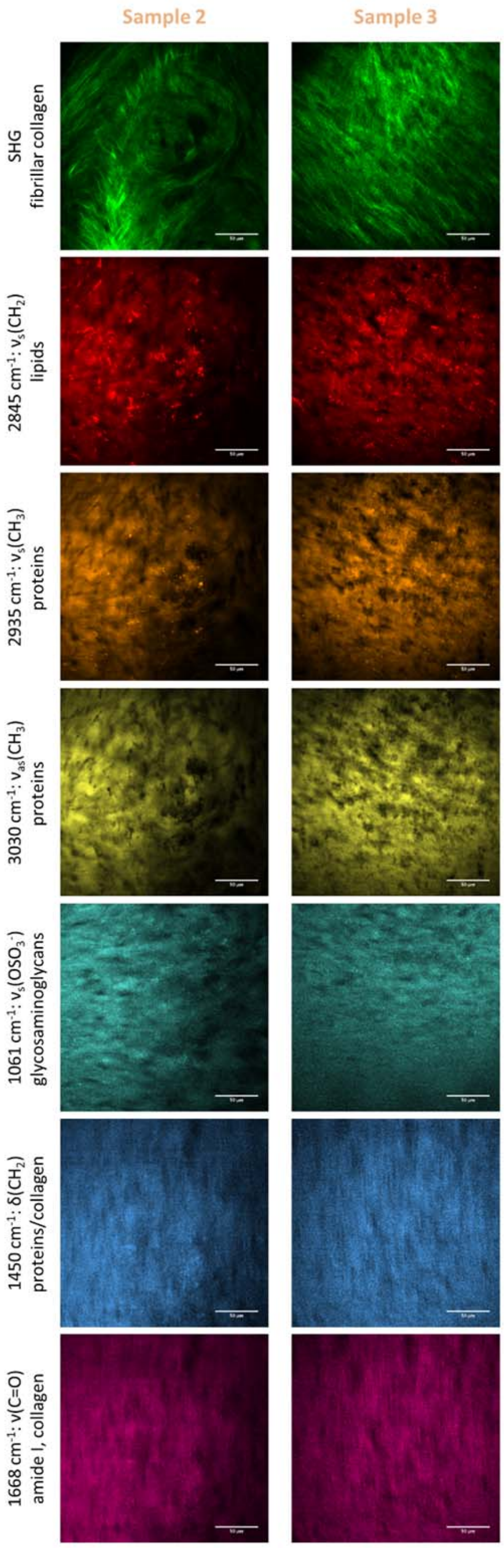

Supplementary Figure 2 – Second harmonic generation (SHG) and coherent anti-Stokes Raman scattering (CARS) images from bioengineered cartilage tissue (replicate samples). Human fetal femur-derived skeletal cells were cultured in an *in vitro* three-dimensional pellet culture system over 21 days in chondrogenic media to differentiate into chondrocytes and generate cartilage tissue.

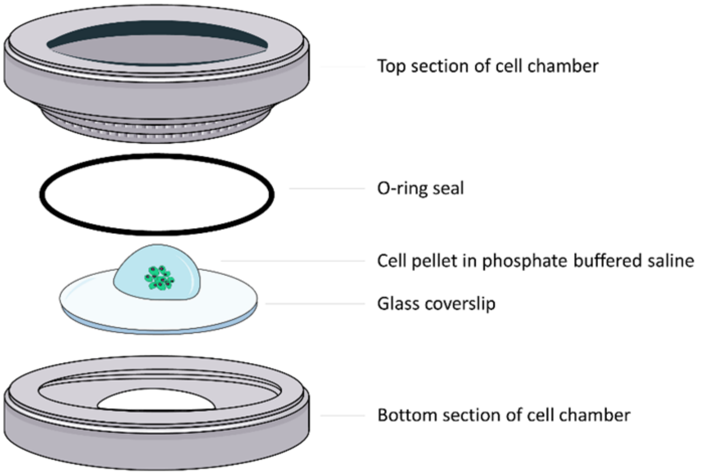

Supplementary Figure 3 – Schematic of the coverslip cell chamber used for live-cell imaging on the multimodal inverted microscope.

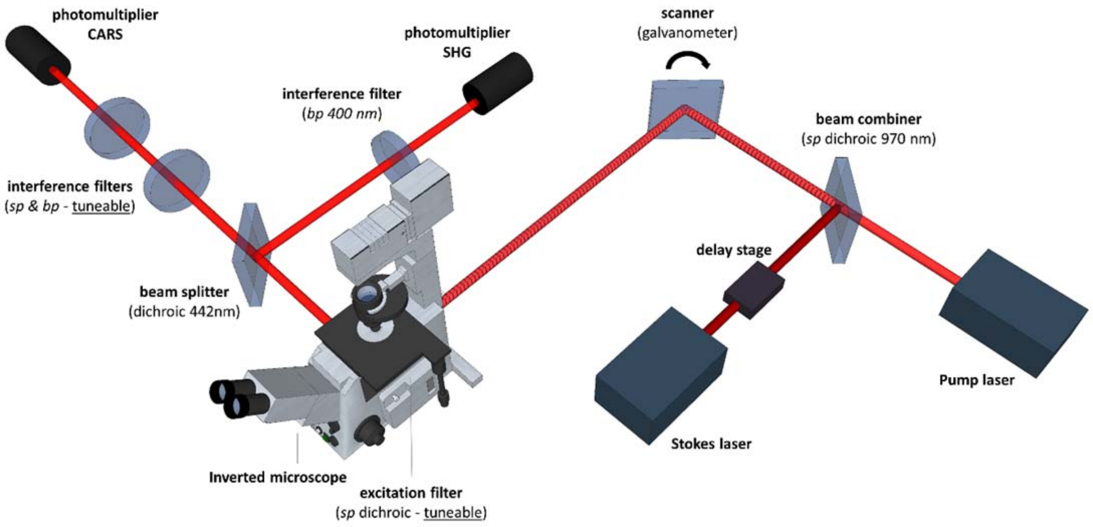

Supplementary Figure 4 – Schematic of the home-built multimodal imaging setup. This multimodal image setup was used for 3D live-cell imaging with simultaneous acquisition of coherent anti-Stokes Raman scattering (CARS) and second harmonic generation (SHG). The pump laser, excitation filter (short pass dichroic) and interference filters (short pass and band pass) are tuneable. For CARS imaging at different wavenumbers we have modified the set up according to Supplementary Table 2.

Supplementary Table 1 – Vibrational modes targeted for CARS imaging and the corresponding modifications in the multimodal label-free imaging set up.

Raman peak	Vibrational assignment	Pump laser	Excitation filter	Interference filters	
			short pass dichroic	*short pass*	*band pass*
1061 cm^{-1}	$\nu_s(OSO_3^-)$	930.2 nm	875 nm	no filter	840/12 nm
1450 cm^{-1}	$\delta(CH_2)$	897.7 nm	825 nm	800 nm	800/12 nm
1668 cm^{-1}	$\nu(C{=}O)$	880.4 nm	825 nm	800 nm	766/13 nm
2845 cm^{-1}	$\nu_s(CH_2)$	797.8 nm	750 nm	775 nm	643/20 nm
2935 cm^{-1}	$\nu_s(CH_3)$	792.1 nm	750 nm	775 nm	643/20 nm
3030 cm^{-1}	$\nu_{as}(CH_3)$	786.2 nm	750 nm	775 nm	643/20 nm

Supplementary Table 2 – Primers used reverse transcription quantitative polymerase chain reaction (qPCR) analysis (F: forward and R: reverse).

Symbol	Transcript	Primer sequence (5’-3’)
ACAN	Aggrecan	F: GACGGCTTCCACCAGTGT<br>R: GTCTCCATAGCAGCCTTCC
ACTB	β-actin	F: GGCATCCTCACCCTGAAGTA<br>R: AGGTGTGGTGCCAGATTTTC
ALPL	Alkaline Phosphatase	F: GGAACTCCTGACCCTTGACC<br>R: TCCTGTTCAGCTCGTACTGC
COL2A1	Alpha-1 Type II Collagen	F: CCTGGTCCCCCTGGTCTTGG<br>R: CATCAAATCCTCCAGCCATC
PPARG	Peroxisome Proliferator Activated Receptor Gamma	F: GGGCGATCTTGACAGGAAAG<br>R: GGGGGGTGATGTGTTTGAACTTG
FABP4	Fatty Acid Binding Protein 4	F: TAGATGGGGGTGTCCTGGTA<br>R: CGCATTCCACCACCAGTT
